# Gender-based pairings influence cooperative expectations and behaviours

**DOI:** 10.1038/s41598-020-57749-6

**Published:** 2020-01-23

**Authors:** Anna Cigarini, Julián Vicens, Josep Perelló

**Affiliations:** 1 OpenSystems Research Group, Departament de Física de la Matèria Condensada, Universitat de Barcelona, Barcelona, 08028 Spain; 2 Universitat de Barcelona Institute of Complex Systems UBICS, Barcelona, 08028 Spain

**Keywords:** Human behaviour, Statistics

## Abstract

The study explores the expectations and cooperative behaviours of men and women in a lab-in-the-field experiment by means of citizen science practices in the public space. It specifically examines the influence of gender-based pairings on the decisions to cooperate or defect in a framed and discrete Prisoner’s Dilemma game after visual contact. Overall, we found that when gender is considered behavioural differences emerge in expectations of cooperation, cooperative behaviours, and their decision time depending on whom the partner is. Men pairs are the ones with the lowest expectations and cooperation rates. After visual contact women infer men’s behaviour with the highest accuracy. Also, women take significantly more time to defect than to cooperate, compared to men. Finally, when the interacting partners have the opposite gender they expect significantly more cooperation and they achieve the best collective outcome. Together, the findings suggest that non verbal signals may influence men and women differently, offering novel interpretations to the context-dependence of gender differences in social decision tasks.

## Introduction

Gender norms and stereotypes play a big role in challenging gender equality and other development goals^1^. Social norms in general, although not always inherently negative, tend to be prejudicial because they are assumptions that disregard a person’s individual and inherent abilities, opportunities and environment. There is indeed a great deal of interest in social norms because of the role that norms can play in reinforcing practices that are seen as problematic^2^. Paying attention to the psychological, social, and cultural influences on decision making and human behaviour can improve the design and implementation of development policies and interventions that target human behaviour^3^. Yet, gender norms and stereotypes are embedded in political, legal, cultural, and economic domains. Since these domains structure access to and control over resources, they also reproduce, strengthen, and legitimate inequitable gender systems^4^. Therefore gender inequity yields critical consequences for how social interactions unfold in public and urban contexts^5–10^.

Although extensive formal literature has developed to explore the context-dependence of social behaviour of men and women, the evidence is mixed^11^. Awareness of the presence of a woman leads single men (but not men in a couple or women) to adopt more cooperative behaviours within their group as a signalling strategy in the context of mate choice^12^. If they are observed by their peer group men lower their prosociality, while women cooperate more often when observed by other women^13^. At the same time, women turn out to be less generous with women, either because of in-group competition or because women might feel weaker or are entitled to less than men^14^. A recent study confirms women’s altruistic behaviour but also shows that both women and men expect women to be more altruistic than men^15^. Yet, another study^16^ found that while women teams seem not to be affected by inter-group competition, men contribute more to their group if their group is competing with other groups. Other contributions however underline that tasks^17^ and context^18^ matter when considering gender differences. A study on gender composition of the team in the workplace found that cooperation between professionals in the operating room tends to increase with a rising proportion of women in clinical team^19^.

All of the many definitions of social norm emphasise the importance of shared expectations, namely the degree of belief, or subjective probability, as for how people should behave^20^. Many social behaviours (within close relationships, organisations, or society at large) would seem irrational (i.e., if individuals did not perform these actions, they would be better off, at least in the short term) without expecting others to behave accordingly^21^. Expectations can directly impact behaviour, and have indeed been taken as a reference point in many behavioural models^22,23^. Likewise, the ability to understand others’ intentions, namely Theory of Mind (ToM)^24^, is as important as the ability to predict their behaviour^25^.

Individuals predict the choices of others based on a set of characteristics, which can be either implicit or voluntary, and which influence social decisions. Arbitrary markers play indeed a key role in solving important coordination problems in human evolution^26,27^. Language^28^ or emotional expressions^29^ also have an impact on individual decision making and collective action. People have been found to cooperate more if they are communicated disappointment rather than anger^30^, or when the partner is smiling^31^. Emotions impact dispute resolution^32^ and punishment behaviour^33^. Humans have also been found to use facial characteristics to make accurate judgements about specific personality traits in others^34^.

No previous study, however, has looked at how non verbal signals influence the behaviour of men and women and their belief about the partner’s intention in ‘hyper-social’ scenarios, namely open and dynamic spaces where diverse individuals continually join, interact and leave (i.e. clubs, informal social gatherings, or public events such as a street arts festival). In hyper-social environments interactions are short-lived, precluding individuals from gaining the necessary experience to make an accurate trust evaluation. Therefore, social decisions might be based on arbitrary signals that favour certain norms of behaviour and not others. This is of particular relevance when it comes to gender norms and stereotypes in public spaces where women’s freedom and enjoyment is sometimes constrained to adhere to restrictive norms for socially acceptable behaviour^35–37^. Does non verbal communication bear different implications for men and women? What do men and women expect from each other in hyper-social environments? And how do such expectations shape men’ and women’s behaviour? We tested the effect of gender-based pairings through non verbal communication on men’ and women’s expectations about their partner’s behaviour and their subsequent willingness to cooperate in a one-shot decision task during a social event in the public space. Moving the laboratory ‘into the wild’^38^ can overcome the excess of seclusion of controlled laboratory settings of much behavioural research. It is also in line with citizen science principles^39,40^ which are increasingly encouraging the opening up of scientific research and practices to the general public^41–49^: from the research design, to the data collection, and the interpretation of the results.

Citizen science can indeed be understood as a form of science that assists the needs and concerns of citizens, and which is developed and enacted by the citizens themselves^50^. However, the fields of the social and behavioural sciences^51^ are still a quite unexplored area under the principles of citizen science. Despite very different perspectives^40,50,52–60^, some authors are therefore advocating for the combination of citizen science principles with social sciences for the research to be applied to, and in line with, societal concerns.

The current study used the discrete symmetric Prisoner’s Dilemma (PD) game to model the interaction between men and women, reducing the complexity of social behaviours to either cooperation or defection. The experiment included two treatments: in the baseline treatment the participants were randomly paired and played anonymously; in the visual treatment the participants were randomly paired and were to stare at each other’s eyes during 30 seconds prior to playing. The unique morphology of the human eye (i.e. white sclera) signifies the special role of gaze behaviour for providing information during social interactions, signalling emotions, and negotiating^61^. The participants self-selected in the hyper-social scenario into the baseline treatment (n = 374), the visual treatment (n = 290), or both (n = 200). Since the aim of the current work is the influence of gender-based pairings on the decision to cooperate or defect in the PD with visual interaction, comments to the samples of participants who played both PDs or to those who played only the PD without visual interaction are made for the seek of comparison. Also, after reading the game instructions (and after visual contact in the visual treatment) participants expectations were elicited by asking them what they expected their partner would do without monetary incentives prior to making their decisions on whether to cooperate or not. This way we could look at whether visual contact influences the beliefs held by participants, and, in turn, their behaviour. Following gaze direction has indeed been correlated with increased cooperation and deception^62,63^. Overall, the results suggest that it is not gender per se that differentiate men and women expectations and behaviour in a hyper-social scenario, but it is rather the gender identity of the partner that contains information which is used differently by men and women to formulate beliefs and subsequent actions. The study focuses on the relationship between expectations and subsequent behavioural outcomes of men and women in ephemeral social interplays, which might provide interesting insights into patterns of men and women interaction in many aspects of their public daily life.

## Results

First, the analysis looks at the expectations that participants have prior to interacting with each others. Expectations play a crucial role in social interactions in that the way humans behave is based on what they think others will do. Gender norms and stereotypes, in particular, are strictly linked to expectations of socially acceptable behaviour. The beliefs that participants hold regarding their partner’s cooperativeness are largely positive (0.85 ± 0.02), with no significant differences between experimental manipulations (see Table 1).

**Table 1:** Probability (mean ± s.e.m (SD)) of cooperation (*p*_*c*_) and expected cooperation (*p*_*ec*_) of the participants who played the Prisoner’s Dilemma without (n = 374) and with (n = 290) visual interaction, and of the participants who played both treatments (n = 200).

	Only one treatment		Both treatments	
	w/o int. (n = 374)	w/int. (n = 290)	w/o int. (n = 200)	w/int. (n = 200)
*p*_*ec*_	0.89 ± 0.02 (0.31)	0.85 ± 0.02 (0.35)	0.89 ± 0.02 (0.32)	0.86 ± 0.02 (0.34)
*p*_*c*_	0.84 ± 0.02 (0.36)	0.84 ± 0.02 (0.37)	0.84 ± 0.03 (0.37)	0.85 ± 0.02 (0.35)

The analysis then look at the aggregate cooperative behaviour of the participants (see Supplementary Tables S1–S3 for detailed information of age and gender distribution of the participants). Since cooperation in social dilemma situations is individually costly, standard economic models predict that people should not cooperate (unless the game is repeated, in which case cooperation might emerge through mechanisms of reciprocity^64,65^). Yet, in the experiment the probability of cooperation greatly differs from self interested predictions (0.84 ± 0.02, Table 1). McNemar’s tests on the reduced sample of participants who played both PDs (n = 200) however did not reveal a significant effect of visual interaction on the cooperation rate (p = 0.7003) nor on expectations of cooperation (p = 0.5563) in the games (Table 1). Given that asking participants to predict the partner’s behaviour might have clearly framed their own behaviour, the high level of expectations may also explain high cooperation rates. Also, the hyper-social scenario within which the experiment took place, namely a street arts festival, might partially account for the large cooperativeness of the participants, which hinders comparisons with previous standard experiments using the PD model.

**Figure 1:**
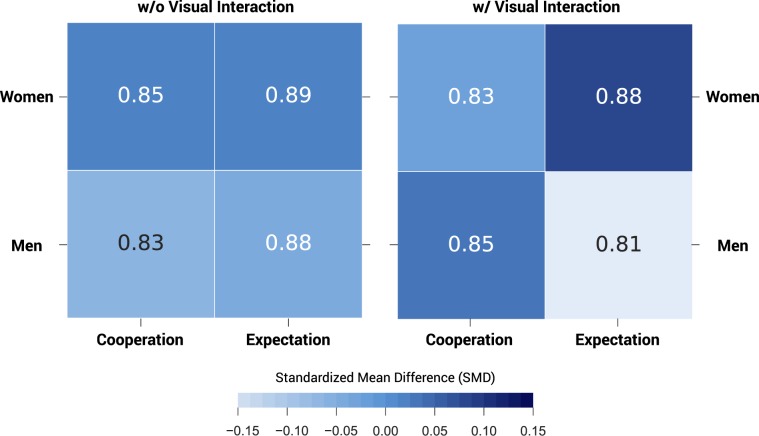
Cooperation and expected cooperation by gender. The heatmap shows the ratio of cooperation and expectation of cooperation by gender in the Prisoner’s Dilemma with (n = 290) and without (n =374) visual interaction. The number in each cell shows the ratio; the colour represents the standardised mean difference, calculated as the difference in means between groups for each behavioural domain divided by the standard deviation of each behavioural domain. See Supplementary Table S5 for further details.

Differences emerge, however, between men and women: when the participants interact visually prior to playing, men display lower expectations regarding the partner’s behaviour compared to women (0.81 ± 0.04 and 0.88 ± 0.02 respectively, p = 0.09166, see Fig. 1 and Supplementary Tables S5 and S14 for further details). Nevertheless, no major differences are found in the levels of cooperation of men and women. Thus, in the PD with visual interaction gender seems to differentiate individual beliefs but not prosocial behaviour. After visual contact men lower their trust in the partner compared to when they interact anonymously (0.81 ± 0.04 and 0.88 ± 0.03 respectively, see Fig. 1).

**Figure 2:**
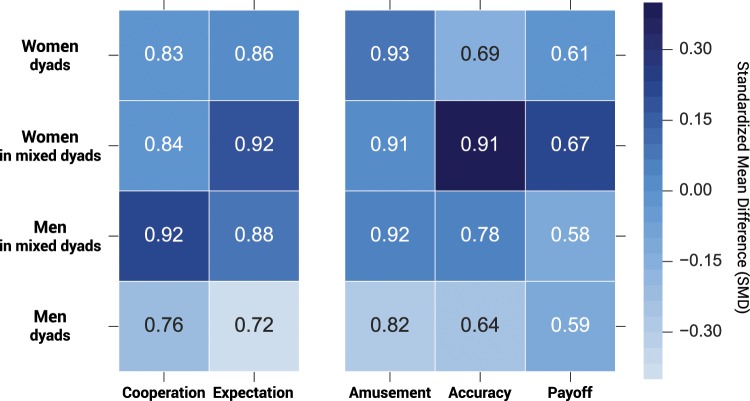
Behavioural domains by gender pairing. *Cooperation* refers to the ratio of cooperative decisions; *Expectation* refers to the ratio of positive expectations regarding the partner’s behaviour, namely cooperation; *Amusement* refers to the ratio of positive arousal following eye contact; *Accuracy* refers to the ratio of correct guesses regarding the partner’s behaviour; *Payoff* refers to the average payoff. The number in each cell shows the normalised value; the colour represents the standardised mean difference, calculated as the difference in means between groups for each behavioural domain divided by the standard deviation of each behavioural domain. See Supplementary Tables S6, S7, S8, and S10 for further details.

To characterise gender differences in expectations and cooperative behaviour, the data set was divided into four groups: women in women dyads (n = 106), women in mixed dyads (n = 67), men in men dyads (n = 50) and men in mixed dyads (n = 67). As summarised in Fig. 2, the results suggest that men’s decreased trust particularly characterises same gender interactions, their expectations being significantly lower if compared to the expectations women hold when interacting with other men (0.72 ± 0.06 and 0.92 ± 0.03 respectively, p = 0.018 see Supplementary Tables S6 and S15). Therefore, it is men dyads who display the most negative beliefs regarding each other’s behaviour. The analysis then looked at the level of amusement the participants felt during visual contact with the partner by asking them whether they enjoyed staring at each others (‘Yes’ or ‘No’) once they played the PD. Indeed, it is men dyads who enjoyed it the least, albeit not significantly (82% ± 5%, see Fig. 2 and Supplementary Table S7 for further details). Thus, eye contact seems to be especially challenging for men’s trust in their same gender peers.

Men dyads diminished expectations translate in lower cooperation rates, which turn out significant when compared to the level of cooperation of men in mixed gender pairs (0.76 ± 0.06 and 0.92 ± 0.03 respectively, p = 0.075 see Fig. 2 and Supplementary Table S6). Women, on the other hand, show rather similar levels of cooperation whether the partner is a man or a woman (0.84 ± 0.05 and 0.83 ± 0.04 respectively, Supplementary Table S6) and do not significantly differentiate their behaviour depending on whom the partner is. Overall, men are significantly more cooperative when they interact with a woman.

If one then compare the elicited beliefs with the actual behaviour of the partner, we find that men and women show different abilities to infer the partner’s behaviour depending on whom the partner is (p = 0.001014, see Supplementary Table S8). The higher cooperation rate of men in mixed dyads is correctly guessed by women 91% (±3%) of the times, which reveals significant differences compared to women in same gender pairs (p = 0.0042) or men in same gender pairs (p = 0.0022, Supplementary Table S8). Therefore, visual contact might benefit women’s accuracy. Indeed, if women get higher payoff than men, on average (1.90 ± 0.06 and 1.75 ± 0.08 respectively, see Supplementary Table S9), it is especially the case for mixed dyads when women’s payoff is the highest (2.01 ± 0.07, see Fig. 2 and Supplementary Table S10 for further details), albeit not significantly.

**Figure 3:**
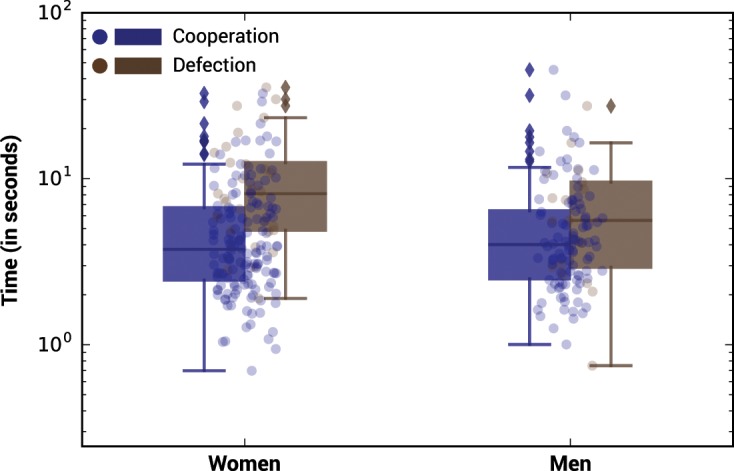
Boxplot of response time by gender. The boxplots represent the log-transformed response time (expressed in seconds) of men’ and women’ cooperation and defection in the Prisoner’s Dilemma with visual interaction. See Table 2 and Supplementary Tables S11, S12, and S13 for further details.

Analysing the response time provides additional insights on the different cognitive processes, or heuristics^66^, that men and women might use to decide whether to cooperate or defect (see Fig. 3). If, overall, cooperative decisions are significantly shorter compared to defecting ones (p = 0.001766, see Supplementary Table S11 for further details), it is particularly the case for women who take significantly more time to defect (10.87s ± 1.60s) than to cooperate (5.47s ± 0.41s) compared to men (see Table 2). The response time of men, on the contrary, becomes statistically indistinct precisely because of visual interaction. However, no relevant variation is found in individual response time in terms of the partner’s gender identity nor in terms of the expectation of the partner’s behaviour (see Supplementary Tables S12 and S13 for further details). Women seem to count on strategic thinking when defecting to a greater extent than men.

**Table 2:** Decision time in seconds (mean ± s.e.m (SD)) in the Prisoner’s Dilemma without (n = 374) and with (n = 290) visual interaction by gender.

		w/o interaction (n = 374)		w/interaction (n = 290)	
		Decision Time (s)	n	Decision Time (s)	n
**Cooperation**	**Women**	5.68 ± 0.33 (4.71)	203	5.47 ± 0.41 (4.98)	144
	**Men**	4.95 ± 0.32 (3.44)	113	5.76 ± 0.61 (6.08)	100
**Defection**	**Women**	9.36 ± 1.13 (6.72)	35	10.87 ± 1.60 (8.62)	29
	**Men**	8.27 ± 1.11 (5.34)	23	7.55 ± 1.60 (6.58)	17
**Aggregated**	**Women**	6.220.34 ± (5.20)^1^	238	6.08 ± 0.46 (6.09)^2^	173
	**Men**	5.51 ± 0.34 (4.00)^3^	136	6.02 ± 0.57 (6.15)	117

The difference in response time between cooperative and defecting decisions is significant for women both in the PD without visual interaction (t = 3.1143, p = 0.003404)^1^ and in the PD with visual interaction (t = 3.2703, p = 0.002582)^2^, and for men only in the PD without visual interaction (t = 2.857, p = 0.008335)^3^.

## Discussion

In summary, the study explored the expectations and social behaviour of men and women in a hyper-social scenario, namely a social event in the public space. It specifically tested the effect of visual contact among interacting partners on the beliefs that participants hold regarding the partner’s intentions and their willingness to cooperate. Overall, the results confirm the context-dependence of gender differences in expectations^15,67^ and decision making^11–14,16,68^ and suggest that visual contact may influence differently the social behaviour of men and women in the public space. It was found that interacting visually with the partner differently affects first order beliefs and subsequent actions of men and women depending on whom they are interacting with. Visual contact is particularly challenging for men’s trust in their same gender peers. Men pairs are indeed the ones with the lowest expectations and cooperation rates. More interestingly, after visual contact women infer men’s behaviour with the highest accuracy. Also, when the interacting partners have the opposite gender they expect significantly more cooperation, and they achieve the best collective outcome.

The fact that, in the experiment, men dyads are more conflicting might be due to different socialisation experiences of men and women: they may internalise different behaviours as a consequence of stereotypes and the different social roles they occupy^66^. Men might develop more agentic skills while women develop more interpersonal skills, and both include their gender stereotype into their self-concept, and self-regulate their behaviour according to these standards^11^. These results reinforce traditional gender stereotypes according to which women are kinder and more cooperative than men. At the same time, uncertainty about social interactions may lead to the evolution of social heuristics^69^, which in turn shape behavioural norms. In line with previous evidence which found that women are expected to be more altruistic^15,67^, in the current study women are expected to be more cooperative compared to men. The fact that women are more intuitively cooperative might also explain why in our study self-interested decisions require women a longer strategic reasoning compared to cooperative decisions^70^.

Men dyads could also be more conflicting because of differential preferences for competitiveness. Extensive work in the laboratory and in the field has indeed shown that men are generally more competitive than women^71^, which may in turn explain observed gender gaps in the labour market. While gender differences in competitiveness might be related to age or the task specificity^17,72^, the cultural setting^72,73^ or the institutional framework^74^, existing evidence shows that competitiveness varies with the gender of the opponent(s)^12,16,18^. In line with the competitive helping hypothesis, according to which men would cooperate more to signal their cooperativeness to women because it might improve their reputation in the context of mate choice^12^, in our work men display higher cooperativeness when interacting with women. This could also explain why men pairs expressed less amusement when asked whether they enjoyed staring at each others’ eyes. They could perceive visual contact more threatening than women^75^. Men have indeed been found to cooperate substantially less often when observed by other men compared to when observed by women, suggesting that men might prefer signalling to other members in their group that they are tough, while women prefer to signal they are inclined to cooperation^13^. Men could thus use cooperation as a signalling strategy, although the lack of data on the participants’ relationship hinders further conjectures.

Women, on the other hand, may use eye contact for ‘mind-reading’^76^, which explains our results on women’s higher accuracy rates. Differences in men’s and women’s ability to assess and predict others’ thoughts, intentions, emotions, and behaviours are indeed a long discussed issue. Overall, women have been found to use strategies related to perspective-taking or ToM reasoning more than men^76,77^, and are also more likely than men to understand others when trying to make a deceptive decision through visual contact^78^. On the other hand, men’s lower accuracy in inferring the intention of the partner also points to a potential misinterpretation of the partner’s signals in hyper-social environments. A heightened ability of women at mind reading is also complemented by a stronger behavioural resilience: their cooperative decisions are not sensitive to the gender of the counterpart.

One can argue that the high cooperation and expectation rates observed might undermine the generalisability of the findings, possibly linked to the little incentives or self-selection biases. Yet, similar experimental settings recently implemented show consistent results^52,79,80^. It is worth to stress, however, that the focus of the current study is on relative differences between men and women, and dyads, rather than absolute values, and on the context-dependence of such relative differences. Therefore, large deviations from self-interested predictions in both conditions (which might be due to the specificity of the experimental context) can, at least partially, account for the lack of a significant effect of the experimental manipulation. Also, while acknowledging the explanatory power of physical or online laboratory settings, the goal was to approximate expectations formation and decision making processes as close as possible to naturalistic interactions^38,53,81^, when the degree of social connectedness between men and women might play a role, and which can hardly be accounted for in unnatural settings where the entire social envelope is altered. In other words, since the primary objective and main contribution of the current work is the analysis of the differential effect of visual contact within and between genders in the public space, self-selection arguments can be ruled out as far as the implications of the findings are constrained to relative differences between men, women and dyads in hyper-social scenarios. Also, given that the focus of the current work was between and within genders (and not between treatments), comparison with the baseline treatment serve for robustness checks and not for the analysis of treatment effect. Finally, although the participants’ compliance to gendered norms of cooperation and defection may follow their specific social preference jointly with the belief that the partner will conform to their expectations (‘empirical expectations’), one cannot conclude anything on whether the participants approve or condemn the opponents’ behaviour since no information was provided on their ‘normative expectations’ (see Methods).

Overall, the evidence on gender differences and the role of stereotypes is mixed. Also, social decisions in hyper-social environments are the results of several complex and interlocking processes. Here, gender pairing appears to be the variable that produces the strongest differences between participants in the study-specific experimental setting. The evidence suggests that it is not gender per se but rather gender pairing that systematically influence the expectations and behaviour of men and women. The study shows significant variations only when the gender of the partner was considered. While the results confirm some gender-typical behaviour, namely men pairs’ lower cooperativeness, they also demonstrate that women can judge the potential of others to cooperate (especially men) at significant rates. Together, the findings shows that non verbal signals influence the beliefs and cooperative behaviours of men and women differently, offering novel interpretations to the context-dependence of gender differences in social decision tasks. More research is needed to determine when and why gender differences in cooperative behaviours and expectations arise. Future studies could test the robustness of our results in different hyper-social scenarios, with varying non-verbal signals, competitive settings and group size, or with varying available information on the gender of participants. Increasing the number of rounds and shifting the decision-space are other two possible modifications to the actual experimental setup. Eventually, if cooperative expectations and behaviours of men and women in the public space depend on non-verbal signals, policies aimed at promoting gender equality should focus on the role that belief systems and expectations play in reproducing culturally recognised gender patterns.

## Methods

All participants were fully informed about the purpose, methods and intended uses of the research. No participant could approach any experimental station without having signed a written informed consent. The use of pseudonyms ensured the anonymity of participant’s identity, in agreement with the Spanish Law for Personal Data Protection. No association was ever made between the participant’s real names and the results. The whole procedure was approved by the Ethics Committee of Universitat de Barcelona. All methods were performed in accordance with the relevant guidelines and regulations. The experiment draws on a tested experimental paradigm^79^ running a ‘lab-in-the-field’ experiment which combines elements of both lab and field experiments by using standardised, validated paradigms from the lab in targeting relevant populations in naturalistic settings^52,65,80^. Building on citizen science principles, the study stresses the need to bring the laboratory in daily contexts, where decisions are usually made, to understand the behaviour of men and women *in vivo*^38,53,81^ overcoming the limitations of closed laboratory settings^82^. The experiment was run during the international festival of performing arts FiraTàrrega, which combines street arts, visual and unconventional shows. It took place in the main square of the municipality of Tàrrega (Catalonia, Spain, 16,000 inhabitants), over two full days on September 2017, from 9 am to 7 pm approximately and as part of the opening show urGENTestimar, a performance that blended performative arts with citizen science to engage local people and increase their sense of ownership with the festival (see Supplementary Fig. S4). The festival hosts more than 100,000 people coming from Catalonia, Spain or worldwide. The experimental infrastructure was designed together with a design team in order to guarantee an attractive and functional design of the experiment able to trigger a massive participation of the public. The experiment was aligned to the inhabitants concerns discussed across a number of workshops prior to the beginning of the festival. The two experimental stations (the PDs with and without visual interaction) were placed at the entrances of the plaza (one for each entry) thus separated from each others. Each station consisted of a mobile wall, with one participant per side, and designed to prevent the participants knowing with whom they were interacting with when playing the PDs. The stations were not apart from the crowd, and people could pass by and watch, although the participants could neither talk to each other nor with the crowd. The experiments were run on electronic tablets through a digital platform specifically developed for the experiment^79^ (see Supplementary Figs. S1, S2, and S3). After an introduction page explaining the context of the game and its incentives, the participants had to fill out a brief sociodemographic questionnaire about their gender identity, age and residence location (details are provided in Supplementary Figs. S1, S2, and S3).

### Experimental design

The experiment included two treatments: one baseline treatment without visual interaction, and one visual treatment with the participants staring at each others eyes for 30 seconds prior to playing^61^. One of the experimental station provided the baseline treatment, and the other experimental station provided the visual treatment. At each experimental station the participants played in pairs a 2 × 2 symmetric and discrete PD, choosing either to cooperate (C) or defect (D)^83^. The participants who self-selected into both treatments played with different partners and were informed accordingly. Their sociodemographic profile coincide with the average profile of the festival attendees^84^ and these profiles are not observed to be different in the two treatments (see Supplementary Tables S1, S2, and S3). The participants earned points following the payoff scheme detailed in the Supplementary Information. At the beginning of the game the participants were told that the incentives for participating were two tickets for a performance at FiraTàrrega equivalent to 30 EUR prize. The festival tickets were assigned after a lottery according to the points earned: the more the points, the higher the chances of winning two tickets. There were five lotteries and thus five winners, and each received the equivalent of 30 EUR prize. Whilst the participants self-selected into treatments, they were unaware of which experimental station was associated to what treatment and of the framings of each treatment. Also, the experimental stations were located far from each other (almost 100 meters distance in a crowded environment). This might rule out possible framing or ordering effects on the self-selection to particular treatments. Rather, the participants were incentivized to play both treatments since this would increase their likelihood of winning two festival tickets. The participants beliefs regarding the partner’s behaviour were also elicited prior to deciding whether to cooperate or defect. Despite the controversy on belief elicitation procedures^85,86^, the participants were not financially rewarded if their stated beliefs about their opponent’s choices corresponded to their opponent’s actual behaviour. Specifically, the participants were asked:’What do you think your partner will do?’. Following the conceptualisation of social norms proposed by^20^, we refer here to ‘empirical expectations’, namely first-order beliefs that the partner will cooperate, without touching upon ‘normative expectations’, namely the second-order beliefs that the partner ought to cooperate. At the end of the game the participants were further asked to fill out a feedback question:’Did you enjoy staring at the eyes of the other person?’ (‘Yes’/‘No’). Finally, we have checked for differences in expectations and cooperation among samples and we did not find any statistically significant difference between the expectations and cooperation levels of the participants who played both PDGs, those who played only the PDG with visual interaction and those who played only the PDG without visual interaction (see Supplementary Tables S16 and S17). There are also no differences in cooperation and expectations depending on the order of the play (see Supplementary Table S18).

### Statistical analysis

Overall, 290 participants participated in the experiment with visual interaction, 374 participated in the experiment without visual interaction, among which 200 participated in both treatments. Only the participants who identified themselves as ‘Man’ or ‘Woman’ were included in the analysis. They ranged in age from 14 to over 65 years old, and 59.6% of the participants to the experiment with visual interaction were women (63.6% women in the experiment without visual interaction). See Supplementary Tables S1, S2, and S3 for further details. The results on cooperative behaviour and expectations are expressed as mean ± s.e.m (SD). The effect of visual interaction on the aggregate rate of expectations and cooperation was tested only in the reduced sample of the participants who played both PDs (n = 200) with the McNemar’s test for paired samples^87^. The difference in expectations, cooperation, guess accuracy and arousal between men and women, and within dyads, is estimated with the Fisher exact test^88,89^, a statistical significance test used in the analysis of contingency tables that calculates the exact significance of the hypothesis testing, rather than relying on an approximation. Wilcoxon rank sum pairwise comparisons with Bonferroni corrections for multiple testing were performed to estimate the difference in expectations, cooperation rates, guess accuracy and arousal between dyads. Welch’s unequal variances t-tests^90^ were performed to estimate the difference in response time and payoffs between men and women, and one-way ANOVA to test the difference in response time and payoffs between dyads.

### Access code

Computer code of the experimental digital interface is available at Github https://github.com/CitizenSocialLab/urGENTestimar. Further details about each of the screen is provided at the Supplementary Information.

### Ethical approval and informed consent

The authors declare that all experimental protocols were approved by the Ethics Committee of Universitat de Barcelona. The methods were performed in accordance with the Spanish Law for Personal Data Protection (current Spanish Organic Law on the Protection of Personal Data and the Guarantee of Digital Rights). Informed consent was obtained from all participants.

## Supplementary information

Supplementary Information.

## Data Availability

The behavioural data that supports the findings of this study are available in Zenodo with the identifier, 10.5281/zenodo.1308977.
