# Supplementary material for: Gender-based pairings influence cooperative expectations and behaviours
Source: Sci Rep. 2020 Jan 23;10:1041. doi: 10.1038/s41598-020-57749-6 (PMC6978365; doi:10.1038/s41598-020-57749-6)
Supplement: Supplementary file 1 — Supplementary Information. [file 41598_2020_57749_MOESM1_ESM.pdf]

Supplementary Information

Gender-based pairings influence cooperative expectations and behaviours

Anna Cigarini[1,2], Julián Vicens[1,2], and Josep Perelló[1,2,*]

[1]OpenSystems Research Group, Departament de Física de la Matèria Condensada, Universitat de Barcelona, Barcelona, 08028, Spain
[2]Universitat de Barcelona Institute of Complex Systems UBICS, Barcelona, 08028, Spain
[*]josep.perello@ub.edu

Structure of the game

The participants played a one-shot Prisoner's Dilemma (PD) with one random partner. The participants were told at the beginning of the experiment that the incentives for participating were two tickets for a performance at FiraTàrrega equivalent to 30 EUR prize. The festival tickets were assigned after a lottery according to the points earned: the more the points, the higher the chances of winning two tickets. There were five lotteries, thus five winners, and each received the equivalent of 30 EUR prize. The two treatments were then posing two different hypothetical situations to enhance their participatory experience. The participants were to share two different basic resources (water and bread) with different, but balanced, payoff matrices: one loaf of bread and 5 cl of water corresponded to 25 points. One loaf of bread was equivalent to 5 cl of water and to 25 points. The participants earned 50 points each if they both decided to cooperate; 0 points each if they both decided to defect; and 75 points the one who defected while the other cooperated (who earned 0 points). They had to simultaneously choose whether to share a basic resource (namely cooperate) or keep it for themselves (namely defect). They had to simultaneously choose whether to share a basic resource (namely cooperate) or keep it for themselves (namely defect).
The payoff matrix of the PD without visual interaction (based on sharing water) is:

$$\begin{array}{c} \\ C \\ D \end{array} \begin{array}{c} \begin{array}{cc} C & D \end{array} \\ \begin{pmatrix} 10 & 0 \\ 15 & 5 \end{pmatrix} \end{array}$$

The payoff matrix of the PD with visual interaction between (based on sharing bread) is:

$$\begin{array}{c} \\ C \\ D \end{array} \begin{array}{c} \begin{array}{cc} C & D \end{array} \\ \begin{pmatrix} 2 & 0 \\ 3 & 1 \end{pmatrix} \end{array}$$

Both PDs have an index of cooperation (see Ref. 85 of the main paper) of 1/3, meaning that the temptation to defect and the risk to cooperate is equal in both scenarios. The lower the index of cooperation, the higher the temptation to defect and thus the risk if one opts for cooperation.

Experimental sample

Supplementary Tables S1, S2 and S3 summarise the gender and age distributions of the participants, and Supplementary Table S4 shows the cooperation and expectation rates by age group in Prisoner's Dilemma with visual interaction. The participant's sociodemographic profile coincides with the average profile of the festival attendees (see Ref. 86 of the main paper). Bonferroni corrected p-values did not reveal any significant differences between age groups neither in cooperation nor expectations.

Table S1. Age distribution by gender and dyads of the participants who played the Prisoner's Dilemma with visual interaction (n=290).

	Total	Women	Men	Pairs same gender		Pairs mixed gender	
				Women	Men	Women	Men
**14-19**	51	25 (15%)	26 (22%)	12 (11%)	11 (22%)	13 (19%)	15 (22%)
**20-29**	79	52 (30%)	27 (23%)	32 (30%)	18 (36%)	20 (30%)	9 (13%)
**30-39**	66	35 (20%)	31 (27%)	21 (20%)	9 (18%)	14 (21%)	22 (33%)
**40-49**	47	28 (16%)	19 (16%)	17 (16%)	7 (14%)	11 (16%)	12 (18%)
**50-59**	32	22 (13%)	10 (9%)	17 (16%)	4 (8%)	5 (8%)	6 (9%)
**60+**	15	11 (6%)	4 (3%)	7 (7%)	1 (2%)	4 (6%)	3 (5%)

Table S2. Age distribution by gender and dyads of the participants who played both Prisoner's Dilemmas (n=200).

	Total	Women	Men	Pairs same gender		Pairs mixed gender	
				Women	Men	Women	Men
**14-19**	40	19 (15%)	21 (28%)	8 (11%)	9 (31%)	11 (20%)	12 (26%)
**20-29**	53	39 (31%)	14 (19%)	21 (30%)	10 (34%)	18 (33%)	4 (9%)
**30-39**	45	27 (22%)	18 (24%)	15 (21%)	4 (14%)	12 (22%)	14 (30%)
**40-49**	32	21 (17%)	11 (15%)	14 (20%)	4 (14%)	7 (13%)	15 (7%)
**50-59**	20	12 (10%)	8 (11%)	9 (13%)	2 (7%)	3 (6%)	6 (13%)
**60+**	10	7 (6%)	3 (4%)	4 (6%)	-	3 (6%)	3 (7%)

Table S3. Age distribution by gender and dyads of the participants who played the Prisoner's Dilemmas without visual interaction (n=374).

	Total	Women	Men	Pairs same gender		Pairs mixed gender	
				Women	Men	Women	Men
**14-19**	84	39 (16%)	45 (33%)	27 (17%)	25 (45%)	12 (15%)	20 (25%)
**20-29**	93	68 (28%)	25 (18%)	51 (32%)	8 (14%)	17 (21%)	17 (21%)
**30-39**	91	57 (24%)	34 (25%)	40 (26%)	13 (23%)	17 (21%)	21 (26%)
**40-49**	52	40 (17%)	12 (9%)	22 (14%)	5 (9%)	18 (23%)	7 (9%)
**50-59**	37	23 (10%)	14 (10%)	13 (8%)	4 (7%)	10 (13%)	10 (13%)
**60+**	17	11 (5%)	6 (5%)	5 (3%)	1 (2%)	6 (7%)	5 (6%)

Table S4. Probability (p) and standard deviation (sd) of cooperation (c) and expectation (ec) by gender and age group in the Prisoner's Dilemma with visual interaction (n=290).

	p_{ec}			p_c		
	All	Women	Men	All	Women	Men
**14-19** (n=51)	0.92 (0.27)	1.00 (-)	0.85 (0.37)	0.86 (0.35)	0.84 (0.37)	0.88 (0.32)
**20-29** (n=79)	0.90 (0.30)	0.90 (0.30)	0.89 (0.32)	0.87 (0.33)	0.86 (0.34)	0.89 (0.32)
**30-39** (n=66)	0.88 (0.33)	0.97 (0.17)	0.77 (0.42)	0.88 (0.33)	0.94 (0.23)	0.81 (0.40)
**40-49** (n=47)	0.79 (0.41)	0.82 (0.39)	0.74 (0.45)	0.81 (0.40)	0.82 (0.39)	0.79 (0.42)
**50-59** (n=32)	0.75 (0.44)	0.68 (0.48)	0.90 (0.32)	0.72 (0.46)	0.64 (0.49)	0.90 (0.32)
**60+** (n=15)	0.73 (0.46)	0.82 (0.40)	0.5 (0.58)	0.80 (0.41)	0.73 (0.47)	1.00 (-)

Supplementary Results

We here present additional analysis that complement the tables presented in the main paper.

Table S5. Probability (mean±s.e.m (SD)) of cooperation (p_c) and expected cooperation (p_{ec}) in the Prisoner's Dilemma without (n=374) and with (n=290) visual interaction by gender.

	w/o interaction		w/ interaction	
	p_c	p_{ec}	p_c	p_{ec}
**Women**	0.85±0.02 (0.35)	0.89±0.02 (0.31)	0.83±0.03 (0.37)	0.88±0.02 (0.32)
**Men**	0.83±0.03 (0.38)	0.88±0.03 (0.32)	0.85±0.03 (0.35)	0.81±0.04 (0.39)
**Fisher's exact test**	0.6562	0.7325	0.7434	0.09166

Table S6. Probability (mean±s.e.m (SD)) of cooperation (p_c) and expected cooperation (p_{ec}) in the Prisoner's Dilemma with visual interaction by dyads. Bonferroni corrected p-values reveal significant differences in cooperation rates between men in same gender pairs compared to men in mixed gender pairs (p=0.075)[1], and in expectation rates between men in same gender pairs and women in mixed gender pairs (p=0.018)[2].

	p_c	p_{ec}
**Women dyads** (n=106)	0.83±0.04 (0.38)	0.86±0.03 (0.35)
**Women in mixed dyads** (n=67)	0.84±0.05 (0.37)	0.92±0.03 (0.26)
**Men in mixed dyads** (n=67)	0.92±0.03 (0.26)	0.88±0.04 (0.33)
**Men dyads** (n=50)	0.76±0.06 (0.43)	0.72±0.06 (0.45)
**Fisher's exact test**	0.08919[1]	0.02364[2]

Table S7. Level of amusement (mean±s.e.m (SD)) following visual contact with the partner by dyads.

	**Amusement**
**Women dyads** (n=106)	0.93±0.02 (0.25)
**Women in mixed dyads** (n=67)	0.91±0.03 (0.29)
**Men in mixed dyads** (n=67)	0.92±0.03 (0.26)
**Men dyads** (n=50)	0.82±0.05 (0.39)
**Fisher's exact test**	0.1607

Table S8. Accuracy of expectations (mean±s.e.m (SD)) by dyads. Bonferroni corrected p-values are significant for comparisons between women in mixed pairs and men in same gender pairs (p=0.0022)[1], and between women in mixed pairs and women in same gender pairs (p=0.0042)[2].

	**Correct guesses**
**Women dyads** (n=106)	0.69±0.04 (0.46)
**Women in mixed dyads** (n=67)	0.91±0.03 (0.29)
**Men in mixed dyads** (n=67)	0.78±0.05 (0.42)
**Men dyads** (n=50)	0.64±0.07 (0.48)
**Fisher's exact test**	0.001014[1,2]

Table S9. Payoff (mean±s.e.m (SD)) in the Prisoner's Dilemma with visual interaction by gender (maximum payoff is equal to 3).

	**Payoff**
**Women**	1.90±0.06 (0.80)
**Men**	1.75±0.08 (0.82)
**Fisher's exact test**	0.1027

Table S10. Payoff (mean±s.e.m (SD)) in the Prisoners' Dilemma with visual interaction by dyads (maximum payoff is equal to 3).

	**Payoff**
**Women dyads** (n=106)	1.83±0.09 (0.91)
**Women in mixed dyads** (n=67)	2.01±0.07 (0.59)
**Men in mixed dyads** (n=67)	1.75±0.09 (0.75)
**Men dyads** (n=50)	1.76±0.13 (0.92)
**ANOVA F-test**	1.507

Table S11. Decision time in seconds (mean±s.e.m (SD)) in the Prisoner's Dilemma without (374) and with (290) visual interaction. The difference in response time for cooperation and defection is significant in both PDs, without (t=4.1414, p=9.816e-05 [1]) and with visual interaction (t=3.2933, p=0.001766 [2])

	**w/o interaction**		**w/ interaction**	
	**Decision Time (s)**	**n**	**Decision Time (s)**	**n**
**Cooperation**	5.42±0.24 (4.30)	316	5.56±0.35 (5.44)	244
**Defection**	8.93±0.81 (6.18)	58	9.67±1.19 (8.07)	46
**Aggregated**	6.00±0.25 (4.81)[1]	374	6.23±0.36 (6.09)[2]	290

Table S12. Decision time in seconds (mean±s.e.m (SD)) in the Prisoner's Dilemma with visual interaction by dyads.

	**Aggregated**		**Cooperation**		**Defection**	
	**Decision Time (s)**	**n**	**Decision Time (s)**	**n**	**Decision Time (s)**	**n**
**Women dyads**	6.95±0.62 (6.39)	106	6.07±0.59(5.57)	88	11.29±1.97 (8.37)	18
**Women in mixed dyads**	5.46±0.66 (5.43)	67	4.53±0.50 (3.74)	56	10.20±2.83 (9.39)	11
**Men in mixed dyads**	5.59±0.63 (5.15)	67	5.39±0.64 (5.08)	62	8.10±2.64 (5.90)	5
**Men dyads**	6.60±1.03 (7.30)	50	6.37±1.21 (7.45)	38	7.32±2.04 (7.09)	12

Table S13. Decision time in seconds (mean±s.e.m (SD)) according to the expectation of the partner's behaviour in the Prisoner's Dilemma with visual interaction by dyads).

	**Aggregated**		**Expecting Cooperation**		**Expecting Defection**	
	**Decision Time (s)**	**n**	**Decision Time (s)**	**n**	**Decision Time (s)**	**n**
**Women dyads**	6.95±0.62 (6.39)	106	6.44±0.66 (6.34)	91	10.08±1.54 (5.97)	15
**Women in mixed dyads**	5.46±0.66 (5.48)	67	5.43±0.52 (4.08)	62	11.15±6.18 (13.77)	5
**Men in mixed dyads**	5.59±0.63 (5.15)	67	5.46±0.67 (5.17)	59	6.51±1.84 (5.20)	8
**Men dyads**	6.60±1.03 (7.31)	50	5.37±0.63 (3.76)	36	9.74±3.26 (12.18)	14

Table S14. Probability (mean±s.e.m (SD)) of cooperation (p_c) and expected cooperation (p_{ec}) of the participants who played both the Prisoner's Dilemma with and without visual interaction (n=200) by gender.

	**w/o interaction**		**w/ interaction**	
	p_c	p_{ec}	p_c	p_{ec}
**Women**	0.83±0.03 (0.37)	0.90±0.03 (0.31)	0.86±0.03 (0.35)	0.90±0.03 (0.30)
**Men**	0.85±0.04 (0.36)	0.87±0.04 (0.34)	0.85±0.04 (0.36)	0.80±0.05 (0.40)
**Fisher's exact test**	0.8424	0.6477	1	0.05309

Table S15. Probability (mean±s.e.m (SD)) of cooperation (p_c) and expected cooperation (p_{ec}) of the participants who played both the Prisoner's Dilemma with and without visual interaction (n=200) by dyads. There are no significant differences in cooperation and expected cooperation between the treatments with and without visual interaction.

	**w/o interaction**		**w/ interaction**	
	p_c	p_{ec}	p_c	p_{ec}
**Women dyads** (n=71)	0.86±0.04 (0.35)	0.87±0.04 (0.33)	0.84±0.04 (0.36)	0.87±0.04 (0.33)
**Women in mixed dyads** (n=54)	0.80±0.05 (0.41)	0.93±0.04 (0.26)	0.87±0.05 (0.34)	0.94±0.03 (0.23)
**Men in mixed dyads** (n=46)	0.89±0.05 (0.31)	0.89±0.05 (0.31)	0.91±0.04 (0.28)	0.85±0.05 (0.36)
**Men dyads** (n=29)	0.79±0.08 (0.41)	0.83±0.07 (0.38)	0.76±0.08 (0.44)	0.72±0.08 (0.45)

Table S16. Difference testing for the probability (mean±s.e.m (SD)) of cooperation (p_c) and expected cooperation (p_{ec}) of the participants who played both Prisoner's Dilemmas (**both**, n=200) and those who played only the Prisoner's Dilemma without visual interaction (**only w/o**, n=174).

	p_c	p_{ec}
**both**	0.84±0.02 (0.37)	0.89±0.02 (0.32)
**only w/o**	0.85±0.03 (0.36)	0.90±0.02 (0.31)
**Fisher's exact test**	0.8863	0.7432

Table S17. Difference testing for the probability (mean±s.e.m (SD)) of cooperation (p_c) and expected cooperation (p_{ec}) of the participants who played both Prisoner's Dilemmas (**both**, n=200) and those who played only the Prisoner's Dilemma without visual interaction (**only w/**, (n=90).

	p_c	p_{ec}
**both**	0.85±0.02 (0.35)	0.86±0.02 (0.34)
**only w/**	0.82±0.04 (0.39)	0.84±0.04 (0.37)
**Fisher's exact test**	0.3858	0.4758

Table S18. Difference testing for the probability (mean±s.e.m (SD)) of cooperation (p_c) and expected cooperation (p_{ec}) of the participants who played first the Prisoner's Dilemma without visual interaction (**w/o > w/**, n=62) compared to those who played first the Prisoner's Dilemma with visual interaction (**w/ > w/o**, n=138).

	**w/o interaction**		**w/ interaction**	
	p_c	p_{ec}	p_c	p_{ec}
**w/o > w/**	0.79±0.05 (0.4)	0.87±0.04 (0.34)	0.84±0.05 (0.37)	0.85±0.04 (0.35)
**w/ > w/o**	0.86±0.03 (0.34)	0.89±0.03 (0.31)	0.86±0.02 (0.34)	0.87±0.03 (0.34)
**Fisher's exact test**	0.2148	0.6413	0.6682	0.8241

Experimental instructions and digital interface

Figure S1 shows the introductory screen, that informs the participants about the general scope of the experiment and the opportunity to win two tickets for a performance in FiraTàrrega 2017. It also shows the sociodemographic questionnaire that the participant had to fill before starting the experiment. The screens in Figure S2 shows the rules of the PD without visual interaction. Figure S3 displays the rules of the PD with visual interaction. Digital platform was able to identify those participants that did participate in the two modalities. This is an important aspect to have a single list of participants that did participate both in the PD without visual interaction and in the PD with prior visual interaction. In case any participant did not understand the instructions, a researcher was there to clarify doubts and make sure the experimental protocol was followed.

Text in **Fig.S1.a**: 40 hours of silence, presence and community within the public space.

We invite you to get in touch with people you don't know, right before entering the plaza. Through your actions we can better know how we relate with each other. We will ask you to make decisions. This will allow you to earn points. The more the points, the higher the chances of winning two tickets for a performance of FiraTárrega 2017!

Text in **Fig.S1.b**: Which of the followings do you feel best describes your gender identity? [Female] [Male] [Trans] [Other] [I don't want to answer] / How old are you? [14-19] [20-29] [30-39] [40-49] [50-59] [60+] / Where do you live? [In Tàrrega] [Outside Tárrega].

Text in **Fig.S2a**: Imagine you are about to share water with the person with whom you are relating. Are you concerned about water? [YES] [NO].

Text in **Fig.S2b**: There are 20 cl of water to be distributed between you and the person standing on the other side of the station. Be careful: The result depends on your decision as well as on the other person's decision. If YOU choose A and SHE chooses A: 10 cl for you and 10 cl for her. If YOU choose A and SHE chooses B: 0 cl for you and 15 cl for her. If YOU choose B and SHE chooses A: 15 cl for you and 0 for her. If YOU choose B and SHE chooses B: 5 cl for you and 5 cl for her. You have to decide: 1. How do you think the other person is going to distribute the water. 2. How do you want to distribute the water.

Text in **Fig.S2.c**: How do you think the **other person** is going to distribute the water? [A] [B].

Text in **Fig.S2.d**: How do **you** want to distribute the water? [A] [B].

Text in **Fig.S2.e**: You choose: [A] / And she chose [B] / You get 0 cls (0 points) / And she gets: 15 cl (75 points).

Text in **Fig.S3.a**: Imagine you have to share some bread with the person with whom you are relating. Are you concerned that somebody has no daily access to food? [YES] [NO] / Did you enjoy staring at the eyes of the other person? [YES] [NO].

Text in **Fig.S3.b**: There are 4 loaves of bread that are to be shared between you and the person who you stared at. Be careful: The result depends on your decision as well as on the other person's decision. If YOU choose A and SHE chooses A: 2 loaves for you and 2 loaves for her. If YOU choose A and SHE chooses B: 0 loaf for you and 3 loaves for her. If YOU choose B and SHE chooses A: 3 loaves for you and 0 loaf for her. If YOU choose B and SHE chooses B: 1 loaf for you and 1 loaf for her. You have to decide: 1. How do you think the other person is going to share the bread. 2. How do you want to share the bread.

Text in **Fig.S3.c**: How do you think the **other person** is going to share the bread? [A] [B].

Text in **Fig.S3.d**: How do **you** want to share the bread? [A] [B].

Text in **Fig.S3.e**: You choose: [A] / And she chose [B] / You get 0 loaves (0 points) / And she gets: 3 loaves (75 points).

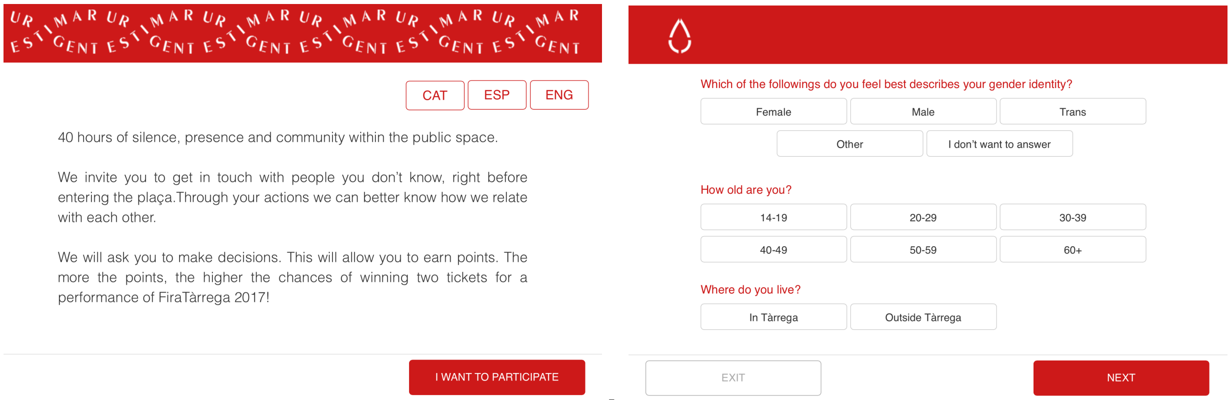

a. b.

Figure S1. Screenshots of the experimental platform. Introductory screen with general information on the research, and the sociodemographic questionnaire.

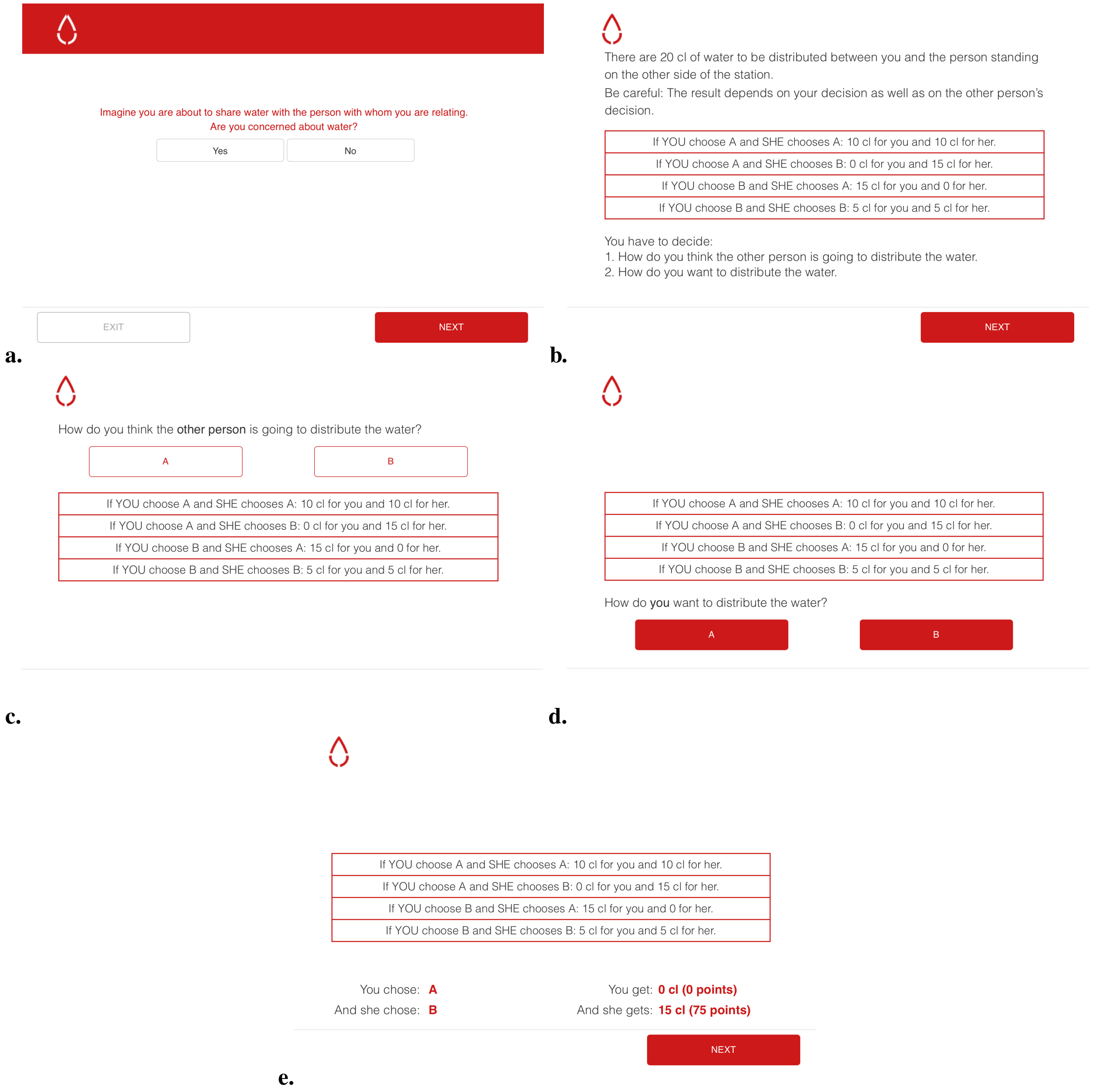

Figure S2. **Screenshots of the experimental platform (Prisoner's Dilemma - water)**. Treatment without visual interaction.

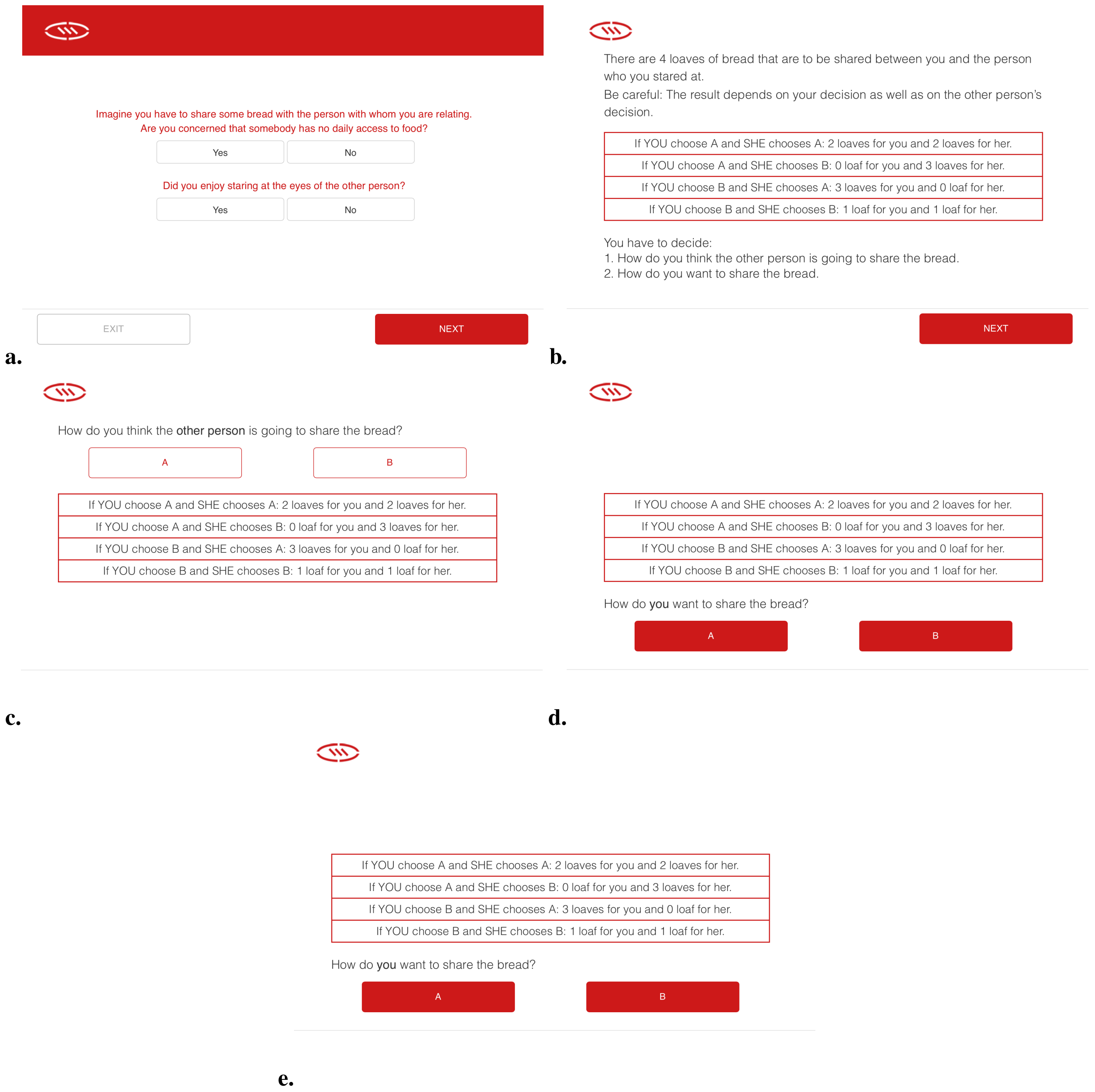

Figure S3. Screenshots experimental platform (Prisoner's Dilemma - bread). Treatment with visual interaction.

Experimental setup

Figure S4 provides a general idea about the experimental setup. It shows the structure of the experimental station (a); and the locations of the two experimental stations at the opposite entrances of the plaza (b), where the opening show *Urgent Estimar* took place. The opening show inaugurated the festival FiraTàrrega 2017 and included the participatory experiment as for the citizen science research we here report. One station allowed for visual interaction among the pairs; the other did not allow for any interaction among the randomly picked pairs of players .

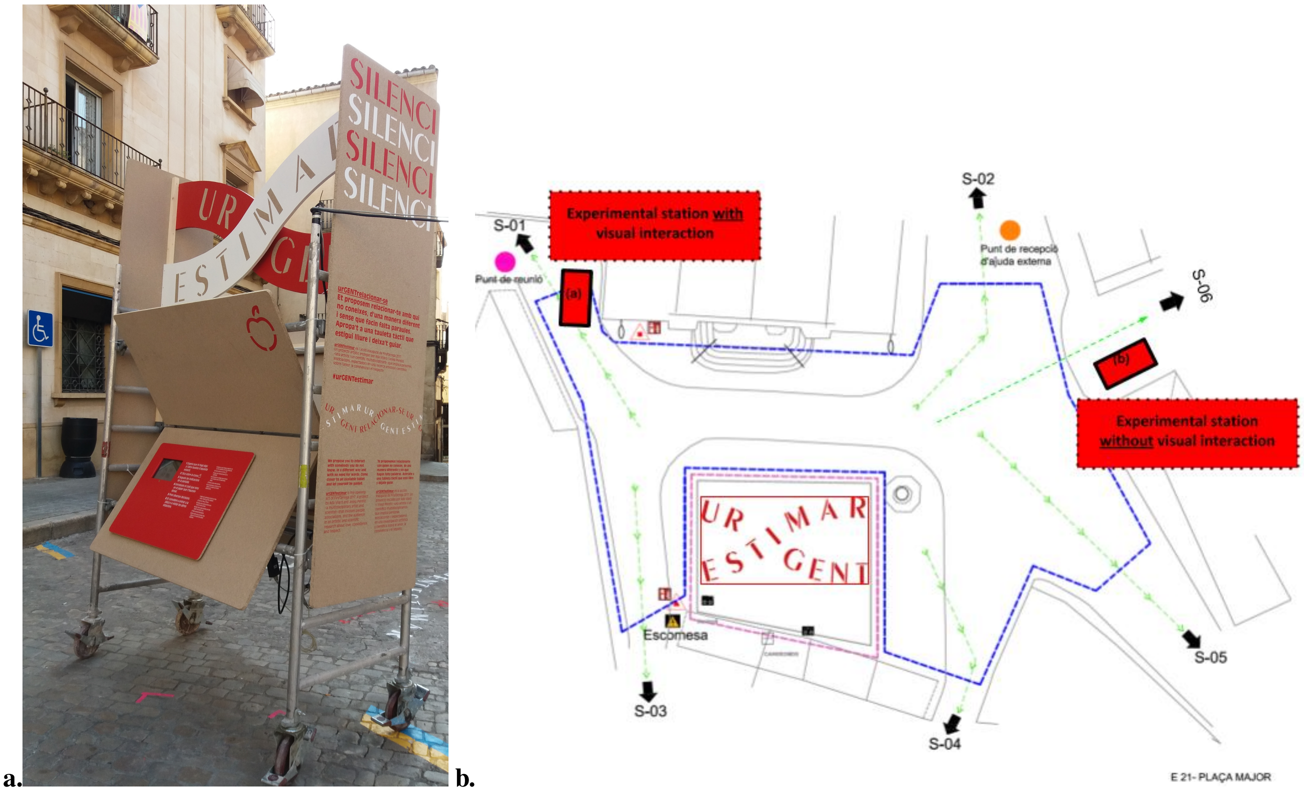

a. b.

Figure S4. **Experimental setup in the hyper social environment**. The images display the structure of the experimental station, and the map of the plaza where the experimental stations were placed and where the show was run. Photograph made by the authors.
